# Expression of the Grape *VaSTS19* Gene in *Arabidopsis* Improves Resistance to Powdery Mildew and *Botrytis cinerea* but Increases Susceptibility to *Pseudomonas syringe* pv Tomato DC3000

**DOI:** 10.3390/ijms18092000

**Published:** 2017-09-17

**Authors:** Yaqiong Wang, Dejun Wang, Fan Wang, Li Huang, Xiaomin Tian, Steve van Nocker, Hua Gao, Xiping Wang

**Affiliations:** 1 State Key Laboratory of Crop Stress Biology in Arid Areas, College of Horticulture, Northwest A&F University, Yangling 712100, China; 18238800765@163.com (Y.W.); 18821713717@163.com (D.W.); wangfan@163.com (F.W.); huanglimakeit@163.com (L.H.); tianxiaomin@163.com (X.T.); 2 Key Laboratory of Horticultural Plant Biology and Germplasm Innovation in Northwest China, Ministry of Agriculture, Northwest A&F University, Yangling 712100, China; 3 Department of Horticulture, Michigan State University, East Lansing, MI 48824, USA; vannocke@msu.edu

**Keywords:** grape, stilbene synthase, resveratrol, powdery mildew, *Botrytis cinerea*, *Pst*DC3000, signal transduction pathways

## Abstract

Stilbene synthase (STS) is a key enzyme that catalyzes the biosynthesis of resveratrol compounds and plays an important role in disease resistance. The molecular pathways linking *STS* with pathogen responses and their regulation are not known. We isolated an *STS* gene, *VaSTS19*, from a Chinese wild grape, *Vitis amurensis* Rupr. cv. “Tonghua-3”, and transferred this gene to *Arabidopsis*. We then generated *VaSTS19*-expressing *Arabidopsis* lines and evaluated the functions of *VaSTS19* in various pathogen stresses, including powdery mildew, *B. cinerea* and *Pseudomonas syringae* pv. tomato DC3000 (*Pst*DC3000). *VaSTS19* enhanced resistance to powdery mildew and *B. cinerea*, but increased susceptibility to *Pst*DC3000. Aniline blue staining revealed that *VaSTS19* transgenic lines accumulated more callose compared to nontransgenic control plants, and showed smaller stomatal apertures when exposed to pathogen-associated molecular patterns (flagellin fragment (flg22) or lipopolysaccharides (LPS)). Analysis of the expression of several disease-related genes suggested that *VaSTS19* expression enhanced defense responses though salicylic acid (SA) and/or jasmonic acid (JA) signaling pathways. These findings provide a deeper insight into the function of *STS* genes in defense against pathogens, and a better understanding of the regulatory cross talk between SA and JA pathways.

## 1. Introduction

Grapevine (*Vitis vinifera* L.) is one of the most agriculturally and economically important fruit species, and is widely cultivated around the world [1]. However, plants are exposed to a large variety of pathogens, such as *Botrytis cinerea* and powdery mildew (*Golovinomyces cichoracearum* UCSC1 (Erysiphe cichoracearum)), as well as hostile environments, including extremes of temperature, light, and water availability. These biotic and abiotic stresses can severely affect the growth, yield, and fruit quality of cultivated grapevine varieties [2]. In order to improve resistance, effective measures need to be taken to prevent or limit pathogen infection and exposure to adverse environments [3]. Natural resistance of plants to phytopathogens is dependent on a large arsenal of constitutive and inducible defense mechanisms [4]. In grapevine, the best characterized defense mechanisms involve synthesis and accumulation of phytoalexins [5,6].

Phytoalexins are low molecular mass, lipophilic antimicrobial compounds that occur in both dicots and monocots [7,8,9]. The stilbenoid phytoalexin, resveratrol, belongs to a major secondary metabolites class derived from the phenylpropanoid pathway, and plays a vital role in plant protection [10,11,12]. Resveratrols are distributed in a taxonomically broad range of plant species among 31 genera and 12 families, including *Fagaceae*, *Liliaceae*, *Moraceae*, *Myrtaceae*, *Papilionaceae*, *Pinaceae*, and *Poaceae*. They have been isolated from various plant structures [13,14]. During the past decade, extensive attention has been given to understanding the biological attributes for plant protection and the biosynthetic regulation of resveratrol [15,16,17].

Stilbene synthase (STS) is a key enzyme responsible for the biosynthesis of resveratrols [18], and condenses three molecules of malonyl-CoA (coenzyme A) and one molecule of coumaroyl-CoA to produce resveratrol [19]. STS has become an area of research focus due to its role in disease resistance. Initial identification and cloning of stilbene synthase gene *(SbSTS1)* from sorghum enabled the transfer of *STS* into a variety of species in order to enhance biotic stress resistance [13,20]. In grapevine, expression of *VaSTS6* significantly improved resistance to powdery mildew [21]. In addition, expression of *STS* in transgenic rice and tobacco plants enhanced resistance to pathogens [22,23].

The grapevine genome contains a family of 32 preliminarily annotated *STS* genes [24], but their precise function and the regulatory mechanism of each individual *STS* gene in response to biotic stresses remains unclear. Previous studies have shown that berries of the Chinese wild grapevine, *V. amurensis* cv.Tonghua-3, express multiple *STS* genes and accumulate resveratrol to high levels during development, and that resveratrol concentration is tightly correlated with expression of a specific *STS* gene, *STS19* [25,26].

The objective of this study was to enhance our understanding of the role of *VaSTS19* gene in biotic stress responses. We found that expression of *VaSTS19* was up-regulated in response to powdery mildew, suggesting that it may be associated with tolerance to biotic stress. We cloned *VaSTS19* and transferred it to *Arabidopsis* to evaluate its role in biotic stress response, specifically in resistance to three pathogenic fungi: powdery mildew, *B. cinerea*, and *Pseudomonas syringae* pv. tomato DC3000 (*Pst*DC3000). Effective defense against pathogens is largely associated with active defense responses regulated by the salicylic acid (SA)- and jasmonic acid (JA)-dependent signaling pathways. Therefore, transcriptional expression profiles of several SA- and JA-related genes were analyzed to assess underlying functions of *VaSTS19* in plant defense. In addition, we analyzed stomatal aperture and callose accumulation in epidermal peels of plants exposed to flagellin fragment (flg22) or lipopolysaccharides (LPS).

## 2. Results

### 2.1. VaSTS19 Expression is Induced in Grape Following Powdery Mildew Infection

**Figure 1:**
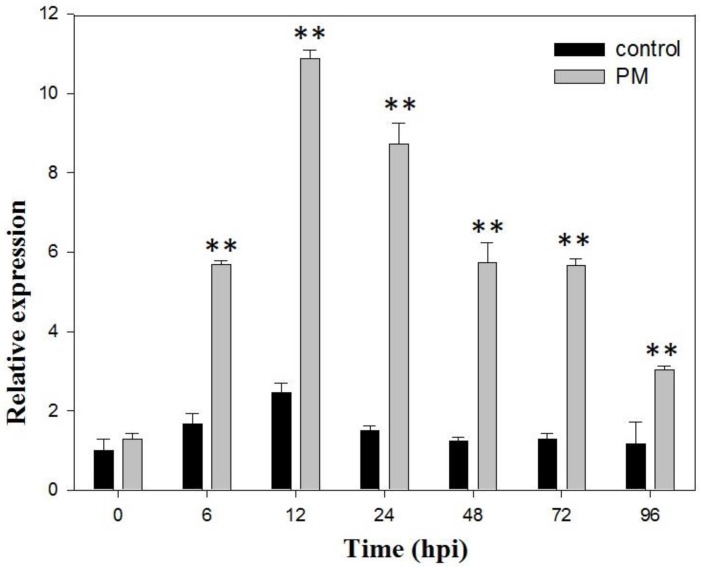
Expression analysis of *VaSTS19* in “Tonghua-3” grapevine plants following powdery mildew infection. Measurements were done using qRT-PCR. Values are the means ± SD from three independent experiments (*n* = 5). Asterisks indicate statistically significant difference (Student’s *t* test, ** *p* < 0.01) between the treated and untreated control plants.

To evaluate expression of *VaSTS19* in response to powdery mildew, quantitative real-time PCR (qRT-PCR) was used to quantify *VaSTS19* transcripts following inoculation of plants with powdery mildew. As shown in Figure 1, *VaSTS19* expression was induced within 6 h upon infection (hours post-infection; hours post-inoculation (hpi)). Expression peaked at 12 hpi, after which it decreased for the next 84 h. As such, we selected the *VaSTS19* gene for all further function analyses in this study.

### 2.2. Analysis of VaSTS19-Expressing Transgenic Arabidopsis Lines

**Figure 2:**
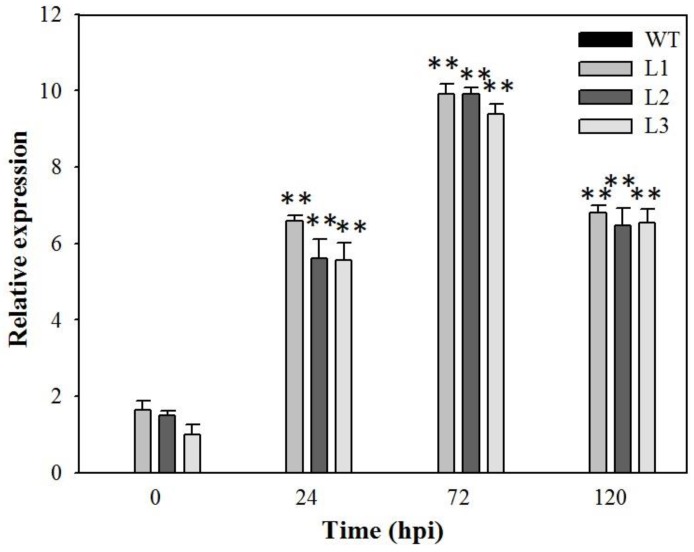
The expression level of *VaSTS19* in transgenic *Arabidopsis* following powdery mildew infection. Measurements were done using qRT-PCR. The lowest expression level in the transgenic plants under non-stressed condition was defined as 1.0. Values are the means ± SD from three independent experiments (*n* = 5). Asterisks indicate statistically significant difference (Student’s *t* test, ** *p* < 0.01) between the expression level of 24, 48, 72, 120 and 0 h after infection.

To evaluate the potential function of *VaSTS19* in powdery mildew resistance in a heterologous system, we first engineered a *VaSTS19* cDNA to be expressed in the plant over-expression vector, pCambia 2300 and introduced this construction into *Arabidopsis*. A total of 50 independent transgenic lines were generated, and the three with the strongest apparent resistance to powdery mildew (designated L1, L2 and L3) were selected to generate homozygous T3 lines, which were used for all further analyses. Measurement of *VaSTS19* expression by qRT-PCR in the three lines following powdery mildew infection (Figure 2) revealed that the transcript levels at 24, 72, and 120 hpi increased 5–10 times when compared with levels at 0 hpi.

### 2.3. Expression of VaSTS19 in Arabidopsis Increases Resistance to Powdery Mildew

**Figure 3:**
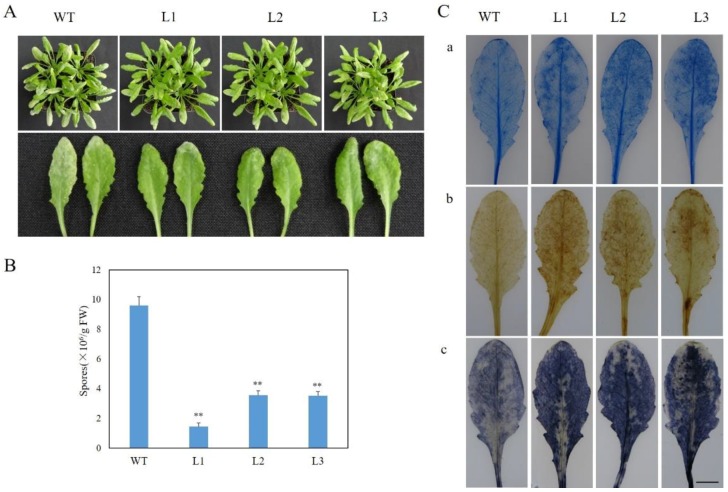
Expression of *VaSTS19* *in Arabidopsis* enhances resistance to powdery mildew. (**A**) Representatives of three transgenic lines and nontransgenic control plants 7 dpi with powdery mildew; (**B**) quantitative analysis of spore numbers on leaves, values are the means ± SD from three independent experiments (*n* = 10), asterisks indicate statistically significant difference (Student’s *t* test, ** *p* < 0.01) between transgenic and nontransgenic lines; (**C**) histochemical staining with trypan blue (**a**) diaminobenzidine (DAB) (**b**) and nitro blue tetrazolium (NBT) (**c**) was performed in order to observe the accumulation of dead cells and reactive oxygen in transgenic and nontransgenic leaves, respectively, and the experiment was repeated three times with 10 leaves each. Scale bars = 10 mm.

To evaluate the phenotypic response of *VaSTS19*-expressing lines to powdery mildew, three transgenic *Arabidopsis* lines and a representative nontransgenic control line were inoculated with powdery mildew, and disease symptoms were monitored. As shown in Figure 3A, nontransgenic plants displayed obvious disease symptoms within seven days post inoculation, with most of infected leaves covered with white powder. In contrast, transgenic plants exhibited only a few leaves with white powder at 7 dpi (Figure 3A). The number of spores per unit weight of leaf was lower in transgenic lines compared to nontransgenic control plants at 5 dpi (Figure 3B). To observe the accumulation of dead cells and active oxygen, we carried out histochemical staining assay with trypan blue, nitro blue tetrazolium (NBT), and diaminobenzidine (DAB). Transgenic plants showed extensive cell death, while nontransgenic plants displayed only fragmented cell death and limited reactive oxygen accumulation on leaves (Figure 3C). These results suggested that expression of *VaSTS19* in *Arabidopsis* enhances resistance to powdery mildew.

### 2.4. Expression Analysis of Disease-Resistance-Related Genes in VaSTS19-Expressing Arabidopsis Lines Following Powdery Mildew Infection

**Figure 4:**
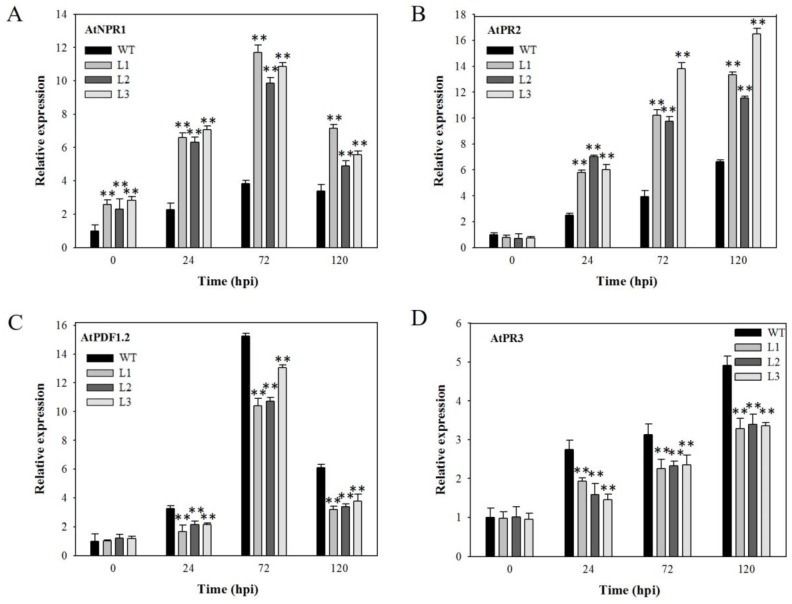
Relative expression levels of disease-resistance-related genes in *VaSTS19*-expressing lines and nontransgenic control plants following powdery mildew infection. The expression levels of (**A**) *AtNPR1*; (**B**) *AtPR2*; (**C**) *AtPDF1.2* and (**D**) *AtPR3*. Measurements were done using qRT-PCR. Values are the means ± SD from three independent experiments (*n* = 5). Asterisks indicate statistically significant difference (Student’s *t* test, ** *p* < 0.01) between transgenic lines and nontransgenic control plants.

SA- and JA-dependent signaling pathways play a large role in defense against pathogens [27]. Therefore, we evaluated the expression levels of four defense-related genes in four-week-old plants at 0, 24, 72, 120 hpi with powdery mildew. We found that the expression of two genes related to SA-mediated defense signaling, *AtNPR1* and *AtPR2*, was markedly increased for both transgenic lines and nontransgenic control plants following powdery mildew inoculation, and showed greater induction in transgenic plants compared to nontransgenic controls (Figure 4A,B). Conversely, the expression of two genes that are actively involved in JA-mediated defense signaling, *AtPR3* and *AtPDF1.2*, was increased at 24, 72, and 120 hpi for both transgenic and nontransgenic plants, but their expression levels in transgenic plants were lower than those in controls at the same point in time following inoculation (Figure 4C,D).

### 2.5. Expression of VaSTS19 in Arabidopsis Enhances Resistance to B. cinerea

**Figure 5:**
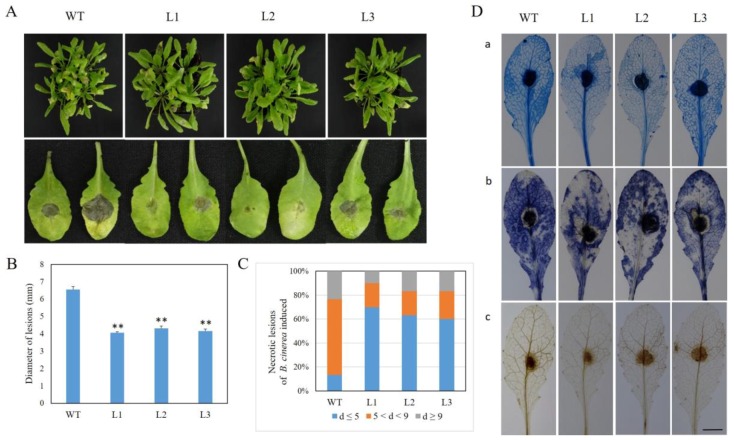
Expression of *VaSTS19* *in Arabidopsis* increases resistance to *B. cinerea* infection. (**A**) Representatives of three transgenic lines and a nontransgenic control line 3 d following *B. cinerea* inoculation; (**B**) average diameter of lesions on leaves of transgenic and nontransgenic lines, data are the means ± SD from three independent experiments (*n* = 30), asterisks indicate statistically significant difference between transgenic and nontransgenic lines (Student’s *t* test, ** *p* < 0.01); (**C**) symptoms of *B. cinerea* 3 dpi were scored by assigning lesions to one of three diameter classes: d ≤ 5 mm; 5 < d < 9 mm; d ≥ 9 mm, and three independent experiments were performed, each with at least 30 leaves; (**D**) transgenic and nontransgenic leaves were stained 3 dpi by trypan blue (**a**), DAB (**b**), and NBT (**c**) to observe the accumulation of dead cells and reactive oxygen, and the experiment was repeated three times, with 10 leaves each. Scale bars = 10 mm.

To assess the function of *VaSTS19* defense to *B. cinerea*, five-week-old plants were inoculated with *B. cinerea* spore suspension. By 48 hpi, we observed necrotizing, water-soaked lesions in both transgenic and nontransgenic lines. By 72 hpi, necrotic symptoms increased in severity in nontransgenic plants. The lesions of transgenic plants were generally less transparent and had a brown color, rather than the black and water-soaked appearance of the control plants (Figure 5A). Lesion size and the proportion of medium and large lesions was lower in transgenic plants (Figure 5B,C). Cell death and basal levels of superoxide were monitored by trypan blue, DAB, and NBT staining in rosette leaves. *VaSTS19*-expressing plants generated lower levels of superoxide and showed lower cell death, in comparison to nontransgenic control plants (Figure 5D). Based on these results, we concluded that *VaSTS19* transgenic lines showed increased defense responses to *B. cinerea*.

### 2.6. Expression Analysis of Disease-Resistance-Related Genes in VaSTS19-Expressing Plants Following B. cinerea Infection

**Figure 6:**
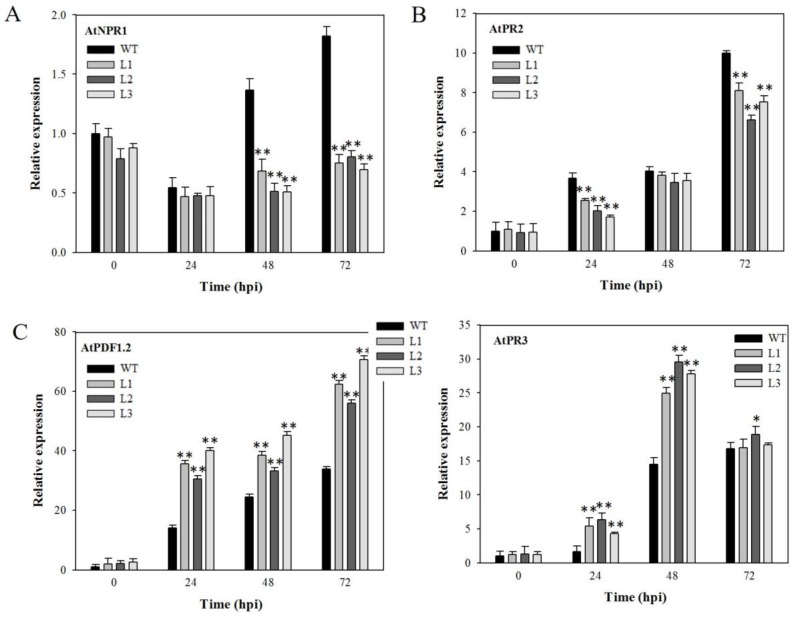
Relative expression levels of four salicylic acid (SA)- and jasmonic acid (JA)-responsive genes in *VaSTS19*-expressing lines and nontransgenic control plants following *B. cinerea* infection. The expression levels of (**A**) *AtNPR1*; (**B**) *AtPR2*; (**C**) *AtPDF1.2* and (**D**) *AtPR3*. Measurements were done using qRT-PCR. Values are the means ± SD from three independent experiments (*n* = 5). Asterisks indicate statistically significant difference (Student’s *t* test, * 0.01 < *p* < 0.05, ** *p* < 0.01) between transgenic lines and nontransgenic control plants.

To elucidate the effect of *VaSTS19* expression on SA- and JA-signal pathways, we monitored the expression of SA- and JA-responsive genes following *B. cinerea* infection at 0, 24, 48, 72 hpi. The expression level of *AtNPR1* dropped in both transgenic and nontransgenic plants at 24 hpi, and then was induced in control plants, but not transgenic lines. From 48 hpi onward, the expression of *AtNPR1* was lower in transgenic lines than in nontransgenic control plants (Figure 6A). We also found that transcript levels of *AtPR2* were induced in both transgenic and nontransgenic plants following *B. cinerea* inoculation, and peaked at 72 hpi. At 24 and 72 hpi, the transcript levels of *AtPR2* in transgenic lines fell to lower levels than in nontransgenic control plants, but there was no difference between transgenic and nontransgenic lines at 48 hpi (Figure 6B). The transcript levels of *AtPDF1.2* and *AtPR3* were also up-regulated following *B. cinerea* infection in both transgenic and nontransgenic control plants and were higher in the transgenic lines compared to nontransgenic control plants (Figure 6C,D). 

### 2.7. VaSTS19-Expressing Arabidopsis Lines Show Decreased Resistance to PstDC3000

**Figure 7:**
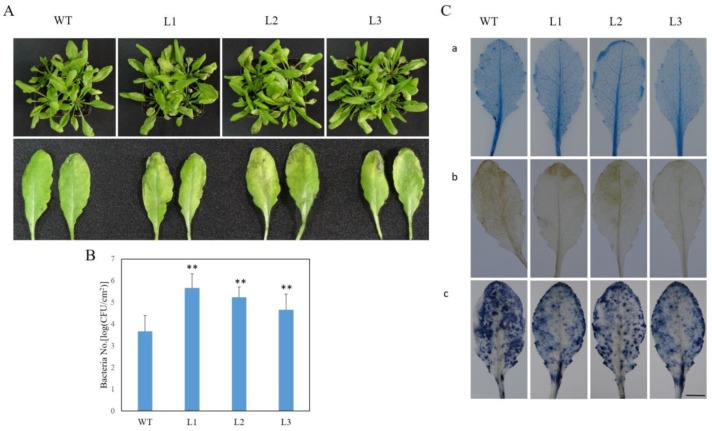
Expression of *VqSTS19* *in Arabidopsis* increases susceptibility to *Pst*DC3000 infection. (**A**) Representatives of three transgenic and nontransgenic lines 3d following *Pst*DC3000 inoculation; (**B**) bacterial numbers per square centimeter on leaves of transgenic and nontransgenic lines at 3 dpi, bars represent the mean ± SD from three independent experiments (*n* = 12), asterisks indicate statistically significant difference (Student’s *t* test, ** *p* < 0.01) between transgenic lines and nontransgenic control plants; (**C**) dead cells and active oxygen production in infected transgenic and nontransgenic lines at 3 dpi using trypan blue (**a**), DAB (**b**) and NBT (**c**) staining, respectively, the experiment was repeated 3 times, with each samples comprising 10 leaves. Scale bars = 10 mm.

To evaluate the effect of *VaSTS19* expression on bacterial pathogen resistance, transgenic and nontransgenic plants were inoculated with *Pst*DC3000. Within 1 dpi, leaves of transgenic lines became yellow and capreolary, and showed yellowish necrosis on surfaces. By 3 dpi, part of the leaves gradually wilted and senesced. Nontransgenic plants showed no apparent disease symptoms although some minor yellowish necrosis was observed (Figure 7A). Results of colony counts revealed that bacterial numbers in transgenic lines were significantly higher compared with in nontransgenic plants (Figure 7B). Staining of infected rosette leaves with trypan blue, DAB, and NBT at 3 dpi revealed small clusters of dead cells and localized superoxide production in transgenic plants, while nontransgenic plants generated high levels of superoxide and showed pervasive cell death (Figure 7C).

### 2.8. Analysis of Disease-Resistance-Related Genes in VaSTS19-Expressing Plants Following PstDC3000 Infection

**Figure 8:**
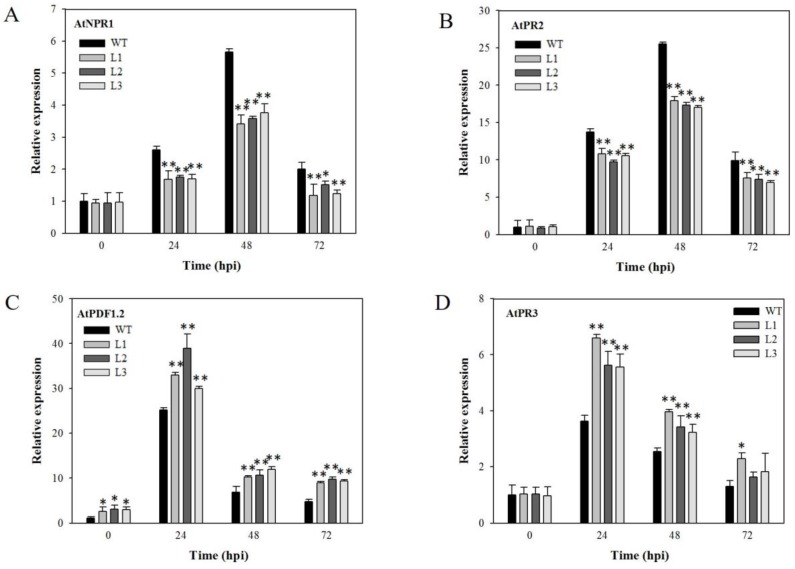
Relative expression levels of disease-resistance-related genes in *VaSTS19*-expressing lines and nontransgenic control plants after *Pst*DC3000 infection. The expression levels of (**A**) *AtNPR1*; (**B**) *AtPR2*; (**C**) *AtPDF1.2* and (**D**) *AtPR3*. Measurements were done using qRT-PCR. Values are the means ± SD from three independent experiments (*n* = 5). Asterisks indicate statistically significant difference (Student’s *t* test, * 0.01 < *p* < 0.05, ** *p* < 0.01) between transgenic lines and nontransgenic control plants.

We also evaluated the expression profile of four SA- and JA-responsive genes in five-week-old plants at 0, 24, 48, and 72 h following *Pst*DC3000 infection. Transcript levels for *AtNPR1* and *AtPR2*, as well as *AtPDF1.2* and *AtPR3*, were increased in both transgenic and nontransgenic lines after *Pst*DC3000 infection, and peaked at 24 or 48 hpi. The expression of *AtNPR1* and *AtPR2* showed an obvious decrease in transgenic lines compared to nontransgenic control plants (Figure 8A,B). Conversely, the expression of *AtPDF1.2* and *AtPR3* was higher in transgenic lines compared to nontransgenic control plants (Figure 8C,D). 

### 2.9. PAMP-Triggered Callose Deposition in VaSTS19-Expressing Lines and Nontransgenic Control Plants

**Figure 9:**
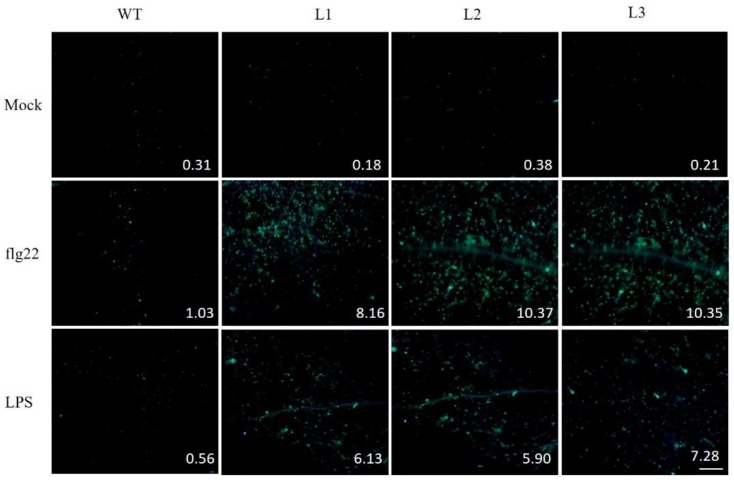
Callose deposition in transgenic and nontransgenic lines following MgSO_4_, flg22, or LPS inoculation. *Arabidopsis* leaves were stained with aniline blue 18 h after infiltration with 10 mM MgSO_4_ (Mock), flg22 (5 μM), or LPS (100 ng/μL). The indicated number on each picture stands for the percent of area deposited by callose in each leaf. This experiment was repeated three times with similar results. Scale bars = 500 μm.

Abundant deposits of the cell wall polymer, callose, are produced as a physical barrier when plants are subjected to biotic challenge [28,29]. We exposed *Arabidopsis* leaves to flg22 or LPS, two distinct pathogen-associated molecular patterns (PAMPs). Aniline blue staining revealed that transgenic lines accumulated a larger amount of callose compared to nontransgenic controls (Figure 9).

### 2.10. Altered Stomatal Immunity Responses in VaSTS19-Expressing Arabidopsis Lines and Nontransgenic Control Plants

**Figure 10:**
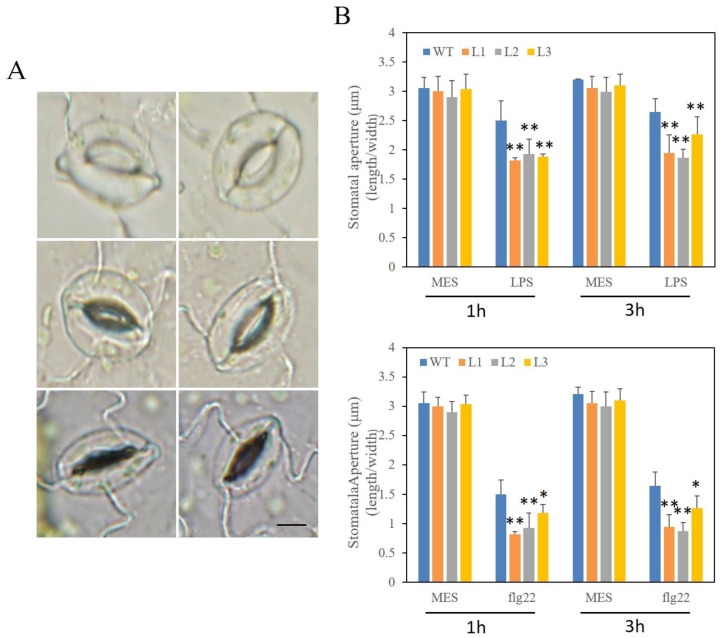
Altered stomatal immunity responses in *VaSTS19*-expressing *Arabidopsis* lines and nontransgenic control plants. (**A**) Open (top and middle panels) and closed (bottom panel) stoma, scale bars = 50 μm; (**B**) stomatal apertures in epidermal peels of transgenic and nontransgenic lines after 1, 3 h of incubation with MES (2-(N-morpholino) ethanesulfonic acid) buffer (Control), flg22 (5 μM) or LPS (100 ng/μL), bars represent the mean ± SD from three independent experiments (*n* = 60), asterisks indicate statistically significant difference between transgenic lines and nontransgenic control plants (Student’s *t* test, * 0.01 < *p* < 0.05, ** *p* < 0.01).

Stomatal closure is part of an integral plant innate immune response to restrict pathogenic bacteria. In order to study the role of *VaSTS19* as a barrier in the PAMP-triggered immunity (PTI) process, we analyzed stomatal aperture in epidermal peels of plants exposed to flg22 or LPS. After 1 h of flg22 or LPS treatment, transgenic lines and nontransgenic controls showed drastically decreased stomatal aperture on the leaf, and this was accompanied by a reduction in the number of open stoma (Figure 10A). The stomatal aperture recovered to the nontreated state in nontransgenic control plants by 3 hpi. On the contrary, stomatal aperture of *VaSTS19*-expressing lines was smaller and remained small at 3 hpi (Figure 10B).

## 3. Discussion

The level of stilbenes in plant tissues is low under optimal growing conditions, but strongly increases to high levels in response to a wide range of pathogens and stresses [30], this increase is mediated by transcription of their biosynthetic genes. The most common stilbene is resveratrol [10], which is catalyzed by *STS*, from one molecule of p-coumaroyl-CoA and three molecules of malonyl-CoA [31]. Based on substrate specificity, *STS* enzymes are often classified into two categories, pinosylvin synthase (PSS) and resveratrol synthase (RS) [32]. Transgenic expression of *STS* has been shown to enhance resistance in a variety of plants, including pea [33], barley [34], papaya [35], white poplar [36], and lettuce [37]. One possible mechanism driving this resistance is the production of resveratrol, as *STS* has been shown to increase resveratrol in diverse plants [37,38].

To better understand the function of *VaSTS19*, the gene was constitutively expressed in the transgenic plants after infection with powdery mildew (Figure 2). Our observation that *VaSTS19*-expressing *Arabidopsis* lines showed improved disease resistance and significant reduction of disease symptoms when challenged with the biotrophic pathogen powdery mildew or necrotrophic pathogens *B. cinerea* (Figure 3A and Figure 5A), is consistent with previous research. For example, transformation of apple with the *Vst1* gene increased general resistance to fungal pathogens [39]. Similarly, transgenic tobacco expressing *STS* showed increased resistance to *B. cinerea* [22]. Jiao et al. also found that an *STS* allele isolated from Chinese wild grapevine (*VPSTS*) conferred resistance to powdery mildew by recruiting salicylic acid signaling [40].

Programmed cell death (PCD) is one of the most common responses to pathogens in plants [28], PCD can decrease resistance to saprophytic fungal pathogens, but can improve the immunity to living biotrophic parasites. In addition, production of reactive oxygen species (ROS) is often associated with cell death in plants [41]. The results of our histochemical staining assays were consistent with phenotypic observations. Transgenic lines displayed more focused and frequent programmed cell death and ROS (H_2_O_2_, O^2−^) accumulation compared to nontransgenic control plants following powdery mildew infection (Figure 3C). In contrast, after *B. cinerea* infection, a decrease in lesions as well as decreased PCD and ROS accumulation was observed (Figure 5B,D). Our results differ from those of previous research, in which expression of *VqSTS21* in transgenic *Arabidopsis* increased susceptibility to *B. cinerea* [42]. Previous reports suggested that the upstream region of *STS* contains important cis-acting elements involved in hormonal and defense response [25]. When plants are exposed to pathogenic fungus, cis-elements can mediate response to activate pathogenesis-related (PR, NPR) proteins to defense different biotic stresses [43,44]. 

Plants live in biotic environments in which they encounter a broad range of pathogens. Therefore, they must possess efficient defense mechanisms to protect themselves from disease. Defense response to pathogens is regulated through two major signaling pathways, an SA-dependent pathway and a JA-dependent pathway [27,45]. In our study, transcript levels of SA- and JA-responsive genes were increased in both transgenic lines and nontransgenic control plants after infection with powdery mildew or *B. cinerea*. Interestingly, *VaSTS19*-expressing *Arabidopsis* lines were found to express several genes related to SA-mediated signaling at significantly higher levels compared with nontransgenic plants in response to powdery mildew (Figure 4), and JA-mediated signaling in response to *B. cinerea* (Figure 6). Previous research has shown that these pathways cross-communicate in an antagonistic or synergistic interaction [46,47,48]. SA and JA act synergistically to induce expression of defense-related genes, and treatment with low concentrations of JA and SA resulted in a synergistic effect on the JA- and SA-responsive genes [49,50]. These findings are consistent with previous studies, and suggest that *VaSTS19* enhances defense responses against powdery mildew and *B. cinerea* though SA and/or JA signaling pathways.

We observed that, in response to *Pst*D3000 infection, leaves of transgenic plants became yellow and capreolary, following yellowish necrosis on surfaces (Figure 7A), and showed greatly reduced peroxide levels and numbers of dead cells (Figure 7B,C). Previous research found that several mutants with defects in SA signaling, including *eds1*, *pad4* (phytoalexin deficient 4), *eds5*, *sid2*, and *npr1*, showed enhanced susceptibility to *Pst*DC3000 [51]. Expression of JA-/ET-inducible transcription factor, *ERF1*, causes enhanced susceptibility to *P. syringae* [52]. In addition, *Pst*DC3000 produces coronatine, [53,54], an important virulence factor for *Pst*DC3000 infection in *Arabidopsis* and tomato plants [55,56]. Our results are in agreement with previous research. *AtNPR1* and *AtPR2*, which are components of the SA signal transduction pathway, as well as *AtPDF1.2* and *AtPR3*, which are JA-responsive marker genes, all exhibited significant increase in transcript levels in both transgenic and nontransgenic plants following *Pst*DC3000 infection. Transcript levels of *AtNPR1* and *AtPR2* were down-regulated in the transgenic lines following *Pst*DC3000 infection (Figure 8A,B), while *AtPDF1.2* and *AtPR3*, were significantly up-regulated (Figure 8C,D). Therefore, we concluded that *VaSTS19* responses to *Pst*DC3000 may be mediated by JA signal transduction pathway, suppressing the SA signal transduction pathway.

Stomatal closure is part of the innate immune response to bacterial invasion. The accumulation of callose and altered stomatal aperture is an important symptom of PAMP-triggered immunity response in plants [57]. Our observation that stomatal aperture returned to an initial state in nontransgenic control plants and was markedly larger than *VaSTS19-*expressing lines (Figure 10), and that transgenic lines accumulated more callose compared to nontransgenic control plants following flg22 or LPS infection (Figure 9), meant that *VaSTS19* play an important role in immunity response and that expression of *VaSTS19* enhanced resistance to biotic stresses.

Here, we studied the role of *VaSTS19* from Chinese wild variety *V. amurensis* cv. “Tonghua-3” in response to plant pathogens through a complex network of signaling pathways, which involve two signaling molecules, SA and JA. Expressing *VaSTS19* in transgenic *Arabidopsis* lines enhanced disease resistance to the biotrophic powdery mildew and the necrotrophic *B. cinerea*, but conversely, increased the susceptibility to semi-biotrophic pathogen *Pst*DC3000. We suggest that SA- and JA-mediated signaling pathways do not function independently, but instead, influence each other through positive and negative regulatory interactions. In short, our research findings may provide insight into the function of *VaSTS19* in plants’ responses to biotic stresses and the signaling pathways that are involved in plant defense against pathogens. Further research should be focused on the mechanisms underlying activation and regulation of defense responses.

## 4. Materials and Methods

### 4.1. Plant Materials and Pathogenic Bacteria

Chinese wild *V. amurensis* cv. Tonghua-3 was grown in the grape germplasm resources orchard of Northwest A&F University, Yangling, Shaanxi, China, and was used for powdery mildew inoculation, and for cloning and expression analysis. Nontransgenic and transgenic *Arabidopsis* lines were grown in a chamber at 21 °C (16 h light, 8 h dark, 100 μmol m^−2^·s^−1^ photon flux density) and 80% relative humidity on a sterile substrate. For all experiments, 4- to 5-week-old plants were used.

Powdery mildew was cultured on *Arabidopsis* pad4 (phytoalexin deficient 4) mutant plants, which were grown in a plant incubator (22 °C, photoperiod 8 h dark/16 h light, light intensity 100 μmol m^−2^·s^−1^) [58]. *B. cinerea* was isolated from tomato and cultured on Potato Glucose Agar medium at 25 °C in the dark. *Pst*DC3000 was grown at 28 °C on King’s B medium containing 50 mg/L rifampicin and 100 mg/L kanamycin [59].

### 4.2. Inoculation with Powdery Mildew in Grape

Fully expanded leaves in the third or fourth position from the shoot tip were infected with powdery mildew through gentle contact with previously infected leaves exhibiting disease symptoms [60]. Newly infected leaves were subsequently collected at 0, 6, 12, 24, 48, 72, and 96 h after inoculation. Leaves sprayed with sterile water at the same time points were used as a control. Samples were immediately frozen in liquid nitrogen, and then stored at −80 °C.

### 4.3. RNA Extraction and Gene Expression Analysis by Quantitative Real-Time PCR

Total grapevine RNA was extracted from powdery mildew-inoculated grapevine leaves, and total *Arabidopsis* RNA was extracted from leaves infected with powdery mildew, *Pst*DC3000 or *B. cinerea*, using the E.Z.N.A.^®^ Plant RNA Kit (Omega Bio-tek, Norcross, GA, USA). First-strand cDNA was synthesized using the PrimeScript^TM^ RTase 1st Strand cDNA Synthesis Kit (TaKaRa Biotechnology, Dalian, China) and diluted 6-fold with sterile water. Quantitative real-time PCR analysis was carried out using SYBR Green (TaKaRa Biotechnology) in a StepOnePlus Real-Time PCR System (Applied Biosystems, Foster, CA, USA), and cycling parameters were: 95 °C for 30 s, and 42 cycles of 95 °C for 5 s, and 60 °C for 30 s. The grape *ACTIN1* gene (GenBank Acc. No. AY680701) or *Arabidopsis ACTIN1* gene (GenBank Acc. No. AT3G18780) was used as an internal control. The sequences of gene-specific primers used for the qRT-PCR reactions are provided in Table 1. Each reaction was performed in triplicate for each of the three biologically replicated sets of cDNA samples. Relative expression levels were analyzed with the IQ5 software using the normalized expression method.

### 4.4. Generation of Transgenic Plants Expressing the VaSTS19 Gene

The *VaSTS19* PCR product described above was cloned into the pGEM^®^-T Easy vector (Promega; Madison, WI, USA). The coding sequence of *VaSTS19* (with *BamH*I and *Sma*I sites at the 5′ and 3′ ends, respectively) was amplified from the pGEM^®^-T Easy-*VaSTS19* vector using gene-specific primers, then inserted downstream of the CaMV 35S promoter in the plant over-expression vector, pCambia 2300 (Cambia, Brisbane, QLD, Australia). Plants were transformed by the floral dip method [61]. T0 seeds were harvested and T1 plants were selected on MS medium (pH = 5.8, 30 g/L sucrose, 7.5 g/L agar) supplemented with 100 mg/L kanamycin [62]. Three lines (L1, L2 and L3) displaying the strongest resistance to powdery mildew were selected from 50 independent lines, and T3 homozygous lines were used for the following research.

### 4.5. Inoculation of Arabidopsis Pathogen

Leaves of 4-week-old plants were inoculated with powdery mildew [63]. Visual scoring of disease phenotypes was performed at 7 dpi. Samples were collected at 0, 24, 72, and 120 hpi, and were used for the analysis of expression of disease-resistance-related genes. Spore counts of susceptible leaves was determined as follows: ten leaf samples were collected from six independent lines, respectively, and weighed and placed in 50 mL tubes containing 40 mL of sterile water and 0.02% of Tween 20, after which the tubes were stirred vigorously for 3 min using a vortex mixer. The resulting spore suspension was diluted 1:10 with 0.02% Tween 20 solution. Spores were then counted by means of a hemocytometer under a dissecting microscope [64].

Conidia from 3-week-old cultured *B. cinerea* were washed, and the spore concentration in the inoculation suspension was adjusted to 10^6^ spores/mL with sterile water. Conidia suspension supplemented with 4% maltose and 1% peptone (*m/v*) was applied to leaves. Inoculated leaves were covered with a plastic lid to ensure a relative humidity of 90–100%. Leaves sprayed with sterile water at the same time served as a control. Samples were collected at 0, 24, 48, and 72 hpi, and were used for the analysis of expression levels of disease-resistance-related genes. Disease incidence and lesion diameter were measured 72 h after inoculation, and the lesion diameter was defined as follows: small (d ≤ 5 mm); medium (5 < d < 9 mm); and large (d ≥ 9 mm) [65].

*Pst*DC3000 was cultured on King’s B medium until an OD_600_ of 0.8 was reached. Bacteria were collected by centrifugation and resuspended in 10 mM MgCl_2_ to a final concentration of 10^8^ cfu/mL. *Arabidopsis* plants were dipped into the cell suspension containing 0.05% Silwet L-77 for 15 min [59] and then placed in a plant incubator under 90% relative humidity until disease symptoms developed. Similar to *B. cinerea* infection, samples were collected at 0, 24, 48 and 72 hpi. For bacterial population assays, 12 infected leaves were obtained from six independent lines at 3 dpi, respectively, and then were sterilized with 70% ethanol for 1 min followed by several rinses. Leaf disks (0.6 × 0.6 cm^2^) were excised and homogenized in 100 mL 10mM MgCl_2_, and then the solution was gradually diluted with sterile water. One hundred microliters of the resulting diluted solution were plated on King’s B agar plates supplemented with 100 mg/mL rifampicin and 100 mg/mL kanamycin, and the plates were incubated in a shaker for 48 h at 28 °C [42].

### 4.6. Histochemical Detection of Cell Death and Superoxide Accumulation

H_2_O_2_ accumulation in leaf tissues was detected by diaminobenzidine (DAB) staining [66]. Inoculated leaves were excised at the base of petiole, placed in 1 mg/mL DAB (Sigma, Beijing, China), and incubated for 8 h in the dark. Subsequently, chlorophyll was removed by incubating in 70% ethanol [58]. For trypan blue staining to detect cell death, infected leaves were soaked in boiled trypan blue solution [67] (a 1:1:1:1:1 ratio of trypan blue, phenol, glycerol, lactic acid, and sterile water) for 5 min, and were subsequently depigmented in 2.5 g/mL chloral hydrate for 1–2 days. For NBT staining, inoculated leaves were incubated in HEPES (4-(2-hydroxyethyl)-1-piperazineethanesulfonic acid) buffer (pH 7.5) containing 6 mM NBT for 2 h with illumination [68].

### 4.7. Observation of Qualitative Stoma and Callose under Biotic Stresses

To assure that most stomata were open before experiments were initiated, plants were kept under light (100 mEm^−2^s^−1^) for at least 3 h [69]. The epidermis was peeled from fully expanded leaves and immediately immersed in MES buffer (25 mM MES-KOH (pH 6.15) and 10 mM KCl), 5 mM flg22 peptide (Flagellin Fragment, Anaspec, Fremont, CA, USA), or 100 ng/μL LPS (lipopolysaccharide, Sigma). Flg22 was dissolved in MES buffer. LPS was dissolved in MES buffer solution containing 0.25 mM MgCl_2_ and 0.1 mM CaCl_2_ [70,71]. At 1 and 3 hpi, samples were placed on glass slides and observed under a microscope. The width and length of the stomatal aperture was measured using the Image-Pro (Olympus Corporation, Tokyo, Japan). For the callose experiment, solution (Mgcl_2_ (10 mM), flg22 (5 mM) or LPS (100 ng/μL)) was injected into rosette leaves using 1 mL needleless syringes, respectively. Leaves were stained with aniline blue (dissolved in 150 mM k_2_HPO_4_ (pH 9.5)) at 18 hpi to detect callose deposition.

### 4.8. Statistical Analysis

All experiments were repeated independently three times for each of the three biological replicates. Results are presented as means and standard errors using Microsoft Excel (Microsoft Corporation, Redmond, WA, USA), SigmaPlot 10.0. Paired *t* tests were performed using the SPSS Statistics 17.0 software (IBM China Company Ltd., Beijing, China).

## 5. Conclusions

Expression of *VaSTS19* in *Arabidopsis* enhanced resistance to powdery mildew and *B. cinerea*, but increased susceptibility to *Pst*DC3000 though SA and/or JA signaling pathways. Aniline blue staining revealed that *VaSTS19* transgenic lines accumulated more callose compared to nontransgenic control plants, and showed smaller stomatal apertures when exposed to pathogen-associated molecular patterns (flg22 or LPS).

## Figures and Tables

**Table 1:** Sequence of oligonucleotide primers used in this study.

Primer Name	Forward and Reverse Primers(5′–3′)	Restriction Enzyme Cutting Site
*VaSTS19*-F1	F:CGGGCTAATTTGAATATGTAA	-
*VaSTS19*-R1	R:CGGTAAGGGTCATAGTGCAGCT	-
*VaSTS19*-F2	F:CGCGGATCCGCGATGGCGTCTGTGGAGGAAA	BamHI site underlined
*VaSTS19*-R2	R:TCCCCCGGGGGATTAGTTGGAATCTGTACCA	XmaI site underlined
*AtNPR1*-F	F:GCTCTGCTCGTCAATGGTTATC	-
*AtNPR1*-R	R:GAGGAGTCGGTGTTATCGGTA	-
*AtPR2*-F	F:CGGTACATCAACGTTGGAA	-
*AtPR2*-R	R:GCGTAGTCTAGATGGATGTT	-
*AtPR3*-F	F:CGCTTGTCCTGCTAGAGGTT	-
*AtPR3*-R	R:GCTCGGTTCACAGTAGTCTGA	-
*AtPDF1.2*-F	F:TCATGGCTAAGTTTGCTTCC	-
*AtPDF1.2*-R	R:AATACACACGATTTAGCACC	-
*AtActin1*-F	F:TCAATCCAGGAGATGTTTAGG	-
*AtActin1*-R	R:ACTGCTGGTACTCTGCGACA	-
*VaActin*-F	F:GATTCTGGTGATGGTGTGAGT	-
*VaActin*-R	R:GACAATTTCCCGTTCAGCAGT	-

## Abbreviations

STS	stilbene synthase
CaMV	cauliflower mosaic virus
pad4	phytoalexin deficient 4
MS	murashige and skoog
CoA	coenzyme A
HEPES	4-(2-hydroxyethyl)-1-piperazineethanesulfonic acid
MES	2-(N-morpholino) ethanesulfonic acid
PstDC3000	*Pseudomonas syringae* pv. tomatoDC3000
SA	salicylic acid
JA	jasmonic acid
hpi	hours post inoculation
dpi	days post inoculation
PCR	polymerase chain reaction
qRT-PCR	quantitative real-time PCR
NPR1	nonexpressor of pathogenesis-related gene
PR2	pathogenesis-related gene 2
PDF1.2	plant defensing 1.2
PR3	pathogenesis-related gene 3
NBT	nitro blue tetrazolium
DAB	diaminobenzidine
LPS	lipopolysaccharides
Flg22	flagellin fragment
PAMP	pathogen-associated molecular pattern
UCSC1	Erysiphe cichoracearum
PCD	programmed cell death
PTI	PAMP-triggered immunity
